# The use of intravenous immunoglobulin in the treatment of Hashimoto’s encephalopathy: case based review

**DOI:** 10.3389/fneur.2023.1243787

**Published:** 2023-09-07

**Authors:** Victoriţa Şorodoc, Mihai Constantin, Andreea Asaftei, Cătălina Lionte, Alexandr Ceasovschih, Oana Sîrbu, Raluca Ecaterina Haliga, Laurenţiu Şorodoc

**Affiliations:** ^1^2nd Internal Medicine Department, Sf. Spiridon Clinical Emergency Hospital, Iasi, Romania; ^2^Internal Medicine Department, Faculty of Medicine, Grigore T. Popa University of Medicine and Pharmacy, Iasi, Romania; ^3^2nd Rheumatology Department, Clinical Rehabilitation Hospital, Iasi, Romania

**Keywords:** Hashimoto’s encephalopathy, autoimmune encephalopathy, anti-thyroid peroxidase, anti-thyroglobulin, intravenous immunoglobulin, alpha-enolase

## Abstract

**Background:**

Hashimoto’s encephalopathy (HE) is a controversial immunological neuropsychiatric disease, with a poorly understood pathogenesis. It is characterized by symptoms of acute or subacute encephalopathy which usually occur in the presence of elevated levels of antithyroid antibodies. Even though it is also known as steroid responsive encephalopathy associated with autoimmune thyroiditis (SREAT), some cases appear to be steroid-resistant. This review examined whether treatment of Hashimoto’s encephalopathy with intravenous immunoglobulin (IVIG) is associated with better clinical outcomes than the standard therapy. Additionally, we presented a case of a 59-year-old man who presented with severe neurological manifestations and was successfully treated with intravenous immunoglobulin.

**Methods:**

The online databases PubMed and EMBASE were searched.

**Results:**

A total of 1,365 articles were identified. After the deletion of 112 duplicates, 1,253 studies were screened by evaluating the title and abstract, focusing on Hashimoto’s encephalopathy cases where IVIG were used. 846 studies were excluded because they were not relevant to the topic or included pediatric population. Therefore, 407 full-text articles were assessed for eligibility. The final analysis included 14 eligible articles after 393 were excluded (irrelevant texts, not written in English, full-text not available). In the majority of the selected case-reports, IVIG was associated with a good outcome, sometimes even with dramatic improvements in patient’s status.

**Conclusion:**

In last years, intravenous immunoglobulin therapy proved its utility in Hashimoto’s encephalopathy’s treatment, being a well tolerated therapy associated with remarkable improvement in patient’s status. Further research is still needed in order to define the optimal treatment protocol for Hashimoto’s encephalopathy and to establish if intravenous immunoglobulin can also be used as a first-line therapy, alone or in combination with steroids.

## 1. Introduction

Hashimoto’s encephalopathy (HE) is a rare autoimmune disease characterized by a variety of neurologic and/or psychiatric symptoms associated with an increase in anti-thyroid antibodies. HE presents a unique diagnostic challenge since the clinical manifestations are often insidious, with cognitive and behavioral disturbance that may associate with tremor, myoclonus or ataxia. Rarely, an acute onset can occur, with manifestations such as stroke-like episodes, epilepsy, or psychosis (1, 2). The term Hashimoto’s encephalopathy was first used in 1966 by Lord Brain for the description of various neurological symptoms in association with Hashimoto’s thyroiditis (3). The cause of HE has been proposed to be autoimmune because of its association with other autoimmune disorders, inflammatory findings in the cerebrospinal fluid (CSF) and response to treatment with steroids. For the severe steroid-resistant HE cases there are only a few reports suggesting that intravenous immunoglobulin (IVIG) might represent a solution.

### 1.1. Clinical case presentation

We report the case of a 59-year-old man, obese, with a history of stage 2 arterial hypertension and chronic venous insufficiency, without any known thyroid disease, who presented with fatigue, tremor, attention deficit, headaches and aphasia. Symptoms started 1 month before presentation, with gradual worsening until he became unable to perform his usual activities of daily living. He had no focal motor, sensory, cranial nerve, or cerebellar abnormalities on physical examination.

An extensive blood workup was performed, with normal results of coagulation tests, liver and kidney function tests, erythrocyte sedimentation rate, C-reactive protein, protein electrophoresis, lactate levels, ammonia levels, tumor markers and viral serology (human immunodeficiency virus, hepatitis B, hepatitis C). A macrocytic anemia associated with a decrease in vitamin B12 levels and presence of gastric parietal cell antibodies was identified.

Thyroid function tests revealed mild hypothyroidism: thyroid stimulating hormone (TSH) titer was 12.6 uIU/ml (normal: 0.4–4.0 uIU/ml); free T4 titer was 0.883 ng/dl (normal: 0.89–1.76 ng/dl) free T3 titer was 3.59 pg/ml (normal: 2.0–4.4 pg/ml). High levels of anti-thyroid antibodies were noted, with anti-thyroid peroxidase (anti-TPO) 657 IU/ml (normal: 0–35 IU/ml) and anti-thyroglobulin (anti-Tg) 629 IU/ml (normal 0–40 IU/ml).

**Figure 1:**
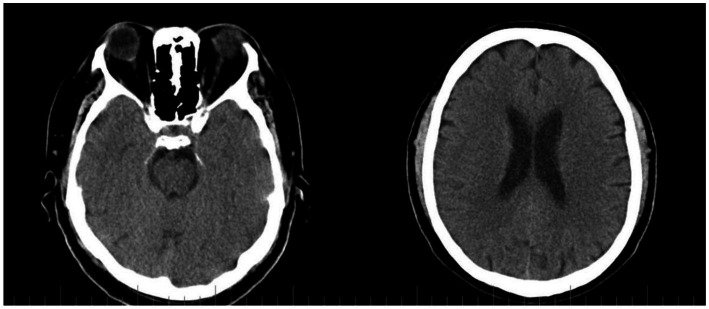
CT scan with no pathological findings.

Cranial computer tomography (CT) was negative for pathologies (Figure 1).

A diagnosis of Biermer anemia and autoimmune thyroiditis was made, with a high suspicion of HE. Intravenous treatment with methylprednisolone 1 g/day was started, associated with levothyroxine and vitamin B12.

After 5 days of therapy the patient experienced a rapidly progressive neurological and psychiatric deterioration, with cognitive dysfunction, confusion, disorientation, visual and auditory hallucinations and paraparesis. Brain magnetic resonance imaging (MRI) detected a moderate atrophy of the fronto-parietal cortex. The patient’s general condition worsened even more, with generalized hypotonia, partial response to pain stimuli and ineffective ventilation, which led to his transfer to intensive care unit. A brain CT scan ruled out a subarachnoid hemorrhage or hematoma, an ischemic stroke or an expansive intracranial lesion. Lumbar puncture revealed elevated levels of proteins in the cerebrospinal fluid.

Because the patient was already on steroids, the response was considered to be unsatisfactory and intravenous immunoglobulin therapy (400 mg/kg daily, for 5 days) was started. Gradual improvement was noticed and a complete recovery developed over the following weeks.

During 1 year follow-up period, remission persisted and the patient was able to perform his usual social activities.

## 2. Materials and methods

In order to summarize the available information regarding the use of IVIG in HE, a literature research was performed in March 2023, using the PubMed and Embase databases, with “Hashimoto encephalopathy” and “intravenous immunoglobulin” as search terms, without any criteria based on the year or type of publication.

After rejecting duplicates, all articles were assessed independently by two authors to rate their quality based on the selection criteria, which included cases where IVIG were used for the treatment of HE. The exclusion criteria were as follows: no relevant content to the purpose of the research; pediatric populations included; not written in English; full-text not available.

## 3. Results

**Figure 2:**
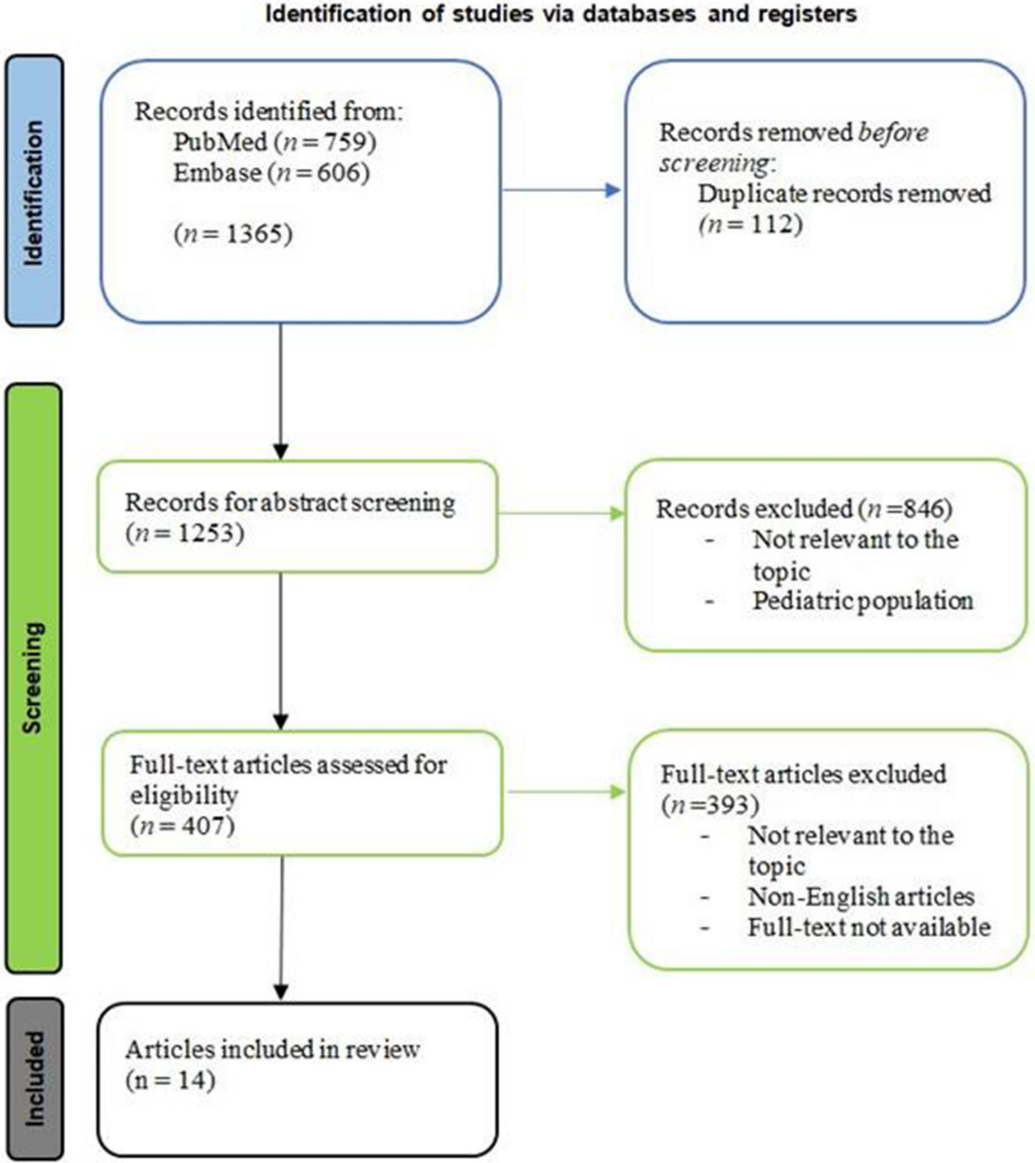
Article selection flowchart, according to PRISMA guideline.

The literature search identified a total of 1,365 articles (Figure 2). After the deletion of 112 duplicates, 1,253 studies were screened by evaluating the title and abstract, focusing on HE cases where IVIG were used. 846 studies were excluded because they were not relevant to the topic or included pediatric population. Therefore, 407 full-text articles were assessed for eligibility. The final analysis included 14 eligible articles after 393 were excluded (irrelevant texts, not written in English, full-text not available; Table 1).

**Table 1:** Schematic description of the selected cases.

Author Ref. # Case	Clinical manifestations	Initial treatment	Response	Secondary treatment	Follow-up
Aladdin et al. (4) 47-year-old ♀	Progressive decline in recent memory, emotional lability, insomnia with agitation, generalized tonic–clonic seizures, post-ictal confusion. Global rigidity, bradykinesia, masked face, anarthria, intermittent choreaoathetotic movements.	i.v. pulse steroid therapy (repeated twice).	Limited.	One course of IVIG.	No sensible changes.
Kondramashin et al. (5) 74-year-old ♂	Progressive cognitive decline over the 6 months preceding presentation. Waxing and waning. confusion which started 24 h prior to presentation.	i.v. solumedrol - 500 mg twice daily, 3 days + IVIG (0,4 mg/kg once daily for 5 days).	Fast improvement in mental status.		
Walia et al. (6) 43-year-old ♀	Declining cognition, confusion, disorientation, inability to communicate or ambulate over the 3 months preceding presentation.	Oral prednisone + IVIG (400 mg/kg/day for 5 days)	Slight improvement of mentation.	IVIG 400 mg/kg/day for 3 days, every 4 weeks + tapering and discontinuation of oral prednisone.	Improvement in mental status and near-resolution of ataxia at 4-months follow-up. The final IVIG cycle was completed after 14 months of treatment. Complete clinical, serological and radiological remission after 7 years.
Ghosh et al. (7) 40-year-old ♀	Progressive mood turnabouts, progressive decline in recent memory, executive dysfunction, dimness of vision over the 8 months preceding presentation. Right-sided focal seizure with secondary generalization at presentation followed by alternating hemiparesis.	i.v. methylprednisolone (1 g/day for 5 days)	No clinical recovery.	IVIG 2 g/kg/day for 5 days.	Complete recovery of sensorium after 5 days of IVIG. Complete resolution of left hemiparesis after 2 weeks. Discharged with oral prednisolone + psychiatric treatment with tapering and discontinuation within the next 3 months.
Algahtani et al. (8) 25-year-old ♀	Gradually progressive dizziness, imbalance, right-hand tremor, difficulty walking 2 months after delivery.	Pulse steroid therapy followed by oral prednisolone.	No significal improvement.	Plasma exchange for 5 days followed by IVIG.	Some improvement of the symptoms. Relapse after 6 months followed by a second round of immunomodulatory therapy.
Laycock et al. (9) 28-year-old ♀	Lethargy, migraines, cognitive decline, poor concentration, generalized muscle aches over 1 year preceding presentation.	Pulsed IVIG therapy (high-dose steroids were avoided because of the concerns raised about the metabolic side effects).	Improvement in general intellectual functioning.		
Zhu et al. (10) 54-year-old ♀	Memory loss and progressive cognitive decline over 5 days preceding presentation. Coma after 4 days.	IVIG for 3 days followed by dexamethasone.	Recovery from coma.		Improvement in memory function at 3 months follow up.
Sapkota et al. (11) 49-year-old ♀	Dizziness, gait imbalance, intermittent slurred speech. Generalized tonic–clonic seizure followed by respiratory failure. Concomittent pulmonary sarcoidosis.	Antiepileptic drug regime.	No clinical improvement.	5 days course of IVIG.	Improvement of cognition and overall encephalopathy after 2 days of IVIG.
He et al. (12) 61-year-old ♂	Cognition dysfunction, slow reaction, impaired short-term memory, postural tremor. One attack of generalized tonic–clonic seizure.	i.v. acyclovir for 7 days + methylprednisolone for 7 days (presumed diagnosis of viral encephalitis).	No important remission of symptoms.	i.v. methylprednisolone 500 mg/day+IVIG 0,4 g/kg/day for 5 days	Improvement of symptoms. Tapering of steroid therapy. Dramatic remission of symptoms at 2 months follow-up.
Drulović et al. (13) 38-year-old ♀	Headache, gait impairment, personality changes, seizures over 6 months before presentation. Known diagnosis of HE which responded well to steroid treatment.	Steroids.	Partial response.	IVIG 0,4 g/kg/day for 5 days.	Complete recovery over the following weeks. Persistence of remission during a 7-year follow-up period.
Cornejo et al. (14) 61-year-old ♀	Malaise and bradypsychia for 2 months followed by a generalized seizure.	i.v methylprednisolone 1 g/day for 5 days followed by prednisone 2 mg/kg/day.	Initial response followed by comatose state.	IVIG 2 g/kg/day for 5 days + prednisone tapering	Resolution of neurological symptoms.
Yuceyar et al. (15) 34-year-old ♀	Impaired attention, memory deficits, aggressive behavior, psychomotor restlessness, palpitations, weight loss, generalized tonic–clonic seizures. Recurrent encephalopathy after each episode of hashitoxicosis.	i.v methylprednisolone 1 g/day for 7 days followed by 60 mg daily oral prednisolone.	Initial clinical improvement followed by seizure recurrence.	IVIG 2 g/kg/day for 3 days.	Improvement of confusional state but persistance of seizures. Multiple relapses in the next period. 3.5 months of 60 mg daily oral prednisolone. 2 months of 100 mg/day oral azathiopirine. 2 rounds of plasmapheresis in 2 months. Thyroidectomy+azathiopirine+levothyroxine-normal cognitive status.
Jacob et al. (16) 29-year-old ♀	Neuro-psihiatric symptoms over a period of 14 years with multiple hospital admissions.	i.v dexamethasone 16 mg/day for 5 days.	Initial response followed by multiple relapses.	IVIG 400 mg/kg/day for 5 days.	Dramatic improvement within 24 h with a full recovery.
Wirkowscki et al. (17) 82 year-old ♀	Changes in mental status, multifocal neurological deficits.	Oral prednisone and methotrexate.	No improvement.	Monthly courses of IVIG.	Clinical improvement.

i.v = intravenous.

## 4. Discussion

Hashimoto’s encephalopathy is a rare and controversial neurological disease associated with autoimmune thyroiditis. It is characterized by unspecific neurological symptoms, such as altered mental status, confusion, cognitive decline, stroke-like episodes, seizures, acute delirium, memory loss, aphasia, myoclonus, ataxia, pyramidal and extrapyramidal signs, dementia, personality changes, hallucinations and delusional thinking. Even though the first case of HE was described in 1966, it still remains a poorly understood disorder (18, 19).

### 4.1. Epidemiology and pathogenesis

The prevalence of HE is estimated at 2.1/100,000 in the adult population but it might actually be higher than expected due to its underdiagnosis, taking into consideration that anti-thyroid antibody testing is not a routine investigation made in patients with neurological symptoms (20). It can affect people of all ages, but most commonly it occurs in the 4th-6th decades of life. It is considered to be a rare entity in the pediatric population. Similar to many autoimmune causes, the incidence is higher in females, with a female-to-male ratio of 4:1, which probably results from the fact that women suffer from Hashimoto’s thyroiditis (HT) 10-20-times more often than men (21, 22).

HT has been reported to associate with other autoimmune diseases, such as vitiligo, alopecia, chronic autoimmune gastritis, celiac disease, type 1 diabetes mellitus, multiple sclerosis, rheumatoid arthritis, Sjogren disease, systemic lupus erythematosus, polymyalgia rheumatica, Addison’s disease, hepatitis C virus related mixed cryoglobulinemia (23). The most frequent associations reported are represented by autoimmune thyroiditis + vitiligo + chronic autoimmune gastritis and autoimmune thyroiditis + polymyalgia rheumatica + chronic autoimmune gastritis. Less commonly, HT is accompanied by additional autoimmune-origin endocrinopathies, thus forming what is known as autoimmune polyendocrine syndromes (APS) (24). Taking into consideration the mentioned potential associated pathologies in HT patients, it stands to reason that HE may also be encountered in the context of other autoimmune disorders.

The pathophysiology of HE remains poorly understood. An autoimmune background is suggested by the fluctuating course of the disease, by the presence of anti-thyroid autoantibodies and also by the good response to corticotherapy (25). The pathogenesis of HE has been attributed to three main mechanisms: immune complex deposition in the brain vessel wall, auto-antibody mediated mechanisms (including antibodies directed against thyroid and also extrathyroid antigens) as well as the toxic effects of some hormones produced as a response to hypothyroidism (26). An initial theory suggested that thyrotropin-releasing hormone (TRH) has toxic effects on the central nervous system. This theory was proposed in 1995 after Ishii et al. observed that a patient developed symptoms similar to those found in HE after intravenous administration of TRH (27). Taking into consideration that at the moment of diagnosis the majority of patients with HE are euthyroid, thyroid hormone dysregulation may not actually have a role in the pathogenesis of HE (28).

Despite the elevated titre of anti-thyroid antibodies, especially of anti-thyroid peroxidase antibodies, found in both serum and cerebrospinal fluid of the majority of patients with HE, a direct pathogenic effect on the central nervous system has still not been confirmed (29). The majority of authors agree with the fact that the levels of anti-thyroid antibodies do not correlate with the severity of the disease and that they should be treated only as a marker of an ongoing autoimmune process (30).

Most authors agree with the fact that HE might be the consequence of an autoimmune vasculitis or immune complex deposition with the subsequent disruption of the cerebral microvasculature (31, 32). This theory was confirmed by biopsy or autopsy. Al-Wafai et al. reported the first case of an angiographically proven vasculitis in a HE patient (33).

In recent years, antibodies against amino terminal domain of α-enolase (NH2-α-enolase Ab) have been identified in the serum and cerebrospinal fluid of patients with HE and they have been proposed as a more reliable marker of HE (34). α-enolase is a multifunctional glycolytic metalloenzyme playing multiple functions which is abundantly expressed in most cells, not only in the brain (35). Circulating antibodies against different epitopes of α-enolase have been identified in multiple patologies, such as rheumatoid arthiritis, systemic lupus erythematosus, membranous nephritis, Behcet’s disease, systemic sclerosis, ulcerative colitis, infectious diseases, different forms of cancer (36). Fujii et al. in a proteomic analysis, concluded that in HE, autoantibodies against amino terminal of α-enolase might be an useful and a more specific diagnostic marker, neither carboxyl terminal nor the mid-region of α-enolase showing any specificity for HE (34). In contrast to their findings, in a more recent study, Mattozzi et al. observed that NH2-α-enolase Ab were found in just 1 patient from 24 patients with HE, which brings into question their utility as a specific marker for HE (37).

Gini et al. conducted a study to determine the target of IgG autoimmune response in HE. They examined the binding of IgG present in the serum and CSF of six HE patients and 15 controls to antigens found in the white matter of the human central nervous system (CNS). The results revealed that CSF IgG from HE patients specifically recognized three spots, identified as aldehyde reductase-I (AKRIAI) and dimethylargininase-I (DDAHI) which was present in 2 isoforms. Immunohistochemistry with anti-DDAHI antiserum showed endothelial cells in the normal human CNS. AKRIAI was found to be widely distributed in neurons and endothelial cells through immunohistochemistry. In the mouse CNS, IgG from HE CSF also immunostained both neuronal and endothelial cells. The presence of these specific autoantibodies in the CSF of HE patients could have significant diagnostic and pathogenetic implications, the autoimmune response against these enzymes leading to vascular and/or neuronal damage (38).

Later on, Benvenga et al. searched for amino acid sequence homologies between α-enolase, DDAHI, AKRIAI and the three classical thyroid autoantigens (TPO, Tg, TSH-receptor), which are also expressed in the CNS. They demonstrated the existance of multiple segments of homology between each CNS-protein and each thyroid antigen, suggesting that cross-reactivity between CNS autoantigens and thyroid autoantigens might contribute to the HE pathogenesis (39).

In another paper, Benvenga et al. wanted to extend their research, looking for additional CNS-expressed proteins homologous to thyroid autoantigens. Using bioinformatic methods to address this hypothesis, from a databank of 46.809 CNS-expressed proteins, they identified 46 proteins that shared homology with TSH-receptor, 27 proteins that shared homology with Tg, and 47 proteins that shared homology with TPO. Some proteins had a single segment of homology and other proteins had multiple segments of homology. The CNS areas where those proteins are expressed match CNS areas where pathological findings were detected at biopsy and/or by neuroimaging in patients in HE. They also mentioned the involvement in other autoimmune disorders of the proteins they found (40).

Endres et al. discussed the clinical considerations when dealing with patients in psychiatry who have schizophreniform or affective syndromes and elevated anti-thyroid antibodies. The primary concern was whether immunotherapy should be considered for patients who do not respond to guideline-based treatment to avoid overlooking HE. Out of the 530 patients analyzed, 91 individuals were identified to have elevated anti-thyroid antibodies. The study suggests that patients with anti-TPO and anti-Tg antibodies exhibited more frequent dysfunction in the blood-cerebrospinal fluid barrier, potentially facilitating the transfer of anti-neuronal antibodies from the bloodstream to the CNS. If these antibodies manage to access the CNS compartment, they may lead to neuronal damage (41). Zhu et al. presented a cases of HE with antibodies to α-amino-3-hydroxy-5-methyl-4-isoxazole-propionic acid receptor 2 (AMPAR2) both in serum and CSF, suggesting that AMPAR2 antibodies are not only met in limbic encephalitis and that high levels of thyroid antibodies can cause immune dysfunction, resulting in the production of anti-AMPAR2 antibodies that have harmful effects on neurons (10). Thus, anti-AMPAR2 antibodies could be considered non-specific antibodies which can occur in HE, but a rigorous differential diagnosis is necessary. Another study made by Takashi et al. aimed to investigate the role of anti-neuronal autoantibodies in HE. Two patients with HE symptoms were studied. Autopsy and laboratory analyses were conducted on patient samples. The absence of CNS vasculitis was found in autopsy, but one patient’s serum contained autoantibodies that reacted with a 36-kDa antigenic protein present in a soluble fraction obtained from human cerebral cortex. These findings suggest a potential association between anti-neuronal autoantibodies and HE pathogenesis (42). Current diagnostic criteria for HE require excluding alternative causes and the absence of anti-neuronal antibodies in CSF. Some published articles in literature show cases of autoimmune encephalitis diagnosed based on specific anti-neuronal antibodies, which also show an association with elevated levels of anti-TPO or anti-Tg antibodies. For example, in a case based literature review made by Matera et al., 6 cases of non-paraneoplastic anti-N methyl D-aspartate receptor encephalitis (anti-NMDArE) which also associated elevated levels of anti-thyroid antibodies were presented (43). Further studies are necessary to determine whether the presence of anti-thyroid antibodies is an incidental finding in autoimmune encephalitis or if they have the capacity to trigger other autoimmune processes, leading to the production of anti-neuronal antibodies.

Lately, there has been a surge of interest in a more aggressive form of Hashimoto’s thyroiditis, accompanied by elevated serum IgG4 levels. IgG4-related disease (IgG4-RD) is a rare autoimmune condition characterized by the excessive production of IgG4 antibodies, leading to chronic inflammation and tissue damage in various organs. While the exact cause of IgG4-RD remains unclear, it is believed to result from an abnormal immune response (44). In IgG4-related thyroid disease, the abnormal immune response results in the infiltration of IgG4-secreting plasma cells into the thyroid tissue, leading to chronic inflammation. This immune attack targets the thyroid cells, causing damage and interfering with their normal function. Over time, this inflammation can lead to the development of Hashimoto’s thyroiditis (45). IgG4-related thyroid disease showed correlations with a younger age group, a higher occurrence in males, higher levels of thyroid autoantibodies, diffuse low echogenicity, and a higher prevalence of subclinical hypothyroidism (46). The first reported case which suggested that the IgG4 fraction might account for the neurological manifestations observed in HE was a 60-year-old male who presented with severe symptoms of HE, exhibiting elevated IgG4 levels in both serum and CSF. The patient responded well to corticotherapy, with a subsequent decrease in serum IgG4 levels, while CSF levels of IgG4 were intermediate (47). The intricate and interconnected nature of autoimmune diseases makes possible the hypothesis that patients with IgG4-related thyroid disease and HE may also experience concurrent IgG4-related autoimmune disorders, such as pemphigus vulgaris and foliaceus, myasthenia gravis, thrombotic thrombocytopenic purpura, chronic inflammatory demyelinating polyneuropathy, autoimmune pancreatitis type 1, IgG4-related cholangiopathy, IgG4-related diseases in the head–neck area, IgG4-related kidney disease, etc. (48, 49).

### 4.2. Clinical manifestations

Hashimoto’s encephalopathy manifests with a wide spectrum of symptoms that mimic a variety of neurological and psychiatric disorders. Presentation also varies considerably, with chronic, subacute, acute or fulminant patterns of an altered mental status (31, 50). Based on previous reports, HE has been classified in two subtypes: a vasculitic type and a diffuse progressive type. The vasculitic type is usually a relapsing form of HE characterized by episodic stroke-like symptoms suggesting a vascular background. The second one is characterized by an insidious onset of symptoms with a significant decline in cognitive functions and memory loss (51, 52).

Laurent et al. in a literature review which included 251 patients with HE, highlighted the variety of symptoms of this disease. At the initial clinical presentation, the following manifestations were found: convulsions (47%), confusion (46%), speech disorder (37%), memory impairment (43%), gait disturbance (27%), persecutory delusions (25%), myoclonus (22%), headaches (16%), coma (15%), depression (12%), isolated progressive memory impairment (11%), isolated psychiatric disorder (10%) (53).

As Laurent et al. did, the majority of case reports also prove that seizures are the most common symptom in patients with HE, many of them being the first manifestion of the disease (4). The type of epileptic presentation may include progressive focal or generalized onset seizures and even new-onset status epilepticus (54). Seizures usually occur more often in children with HE than in adult population. Alink et al. found that seizures were present in 80% of 25 children diagnosed with HE (55). For the majority of epileptic manifestations found in HE, common anticonvulsant therapy alone is usually ineffective. Immunotherapy is necessary for both initial and maintenance therapy of seizures (18).

Rare cases of HE with uncommon manifestations were described in isolated reports. Hwang et al. reported the case of a 56-year-old female who presented with orthostatic myoclonus, a manifestation characterized by multiple muscle fasciculations in the lower extremities that appear immediately upon standing (56). Another publication reported the case of a 32-year-old male who received a diagnosis of HE accompanying optic neuritis (57). Termsarasab et al., reported 2 cases of pure cerebellar ataxia without encephalopathy manifestations in 2 patients diagnosed with HE (58). Akathisia, a very rare occurrence of HE, was found in a patient with HE previously followed up for possible Alzheimer’s disease plus Parkinson’s disease (59). All of these neurological manifestations responded well to immunotherapy.

### 4.3. Diagnosis

Even though the first case of HE was described almost 60 years ago, diagnosis of HE still remains a diagnosis of exclusion. The most recent diagnostic criteria were proposed in 2016 by Graus et al. (60). All of the six criteria have to be met (Table 2).

**Table 2:** Diagnostic criteria of Hashimoto’s encephalopathy.

Encephalopathy with seizures, myoclonus, hallucinations, or stroke-like episodes.
Thyroid disease (usually hypothyroidism) – subclinical or mild overt.
Normal brain MRI or with non-specific abnormalities.
Presence of serum thyroid (thyroid peroxidase, thyroglobulin) antibodies – without a specific cut-off value.
Absence of well characterized neuronal antibodies in serum and CSF.
Reasonable exclusion of alternative causes.

The first diagnostic criteria were proposed by Pschen-Rosin et al. in 1999 (61). One of the criterias was the good response to steroid treatment, but in the last years studies proved that there are many cases of steroid-resistant HE.

Due to the wide variety of conditions that can present with encephalopathy and the symptoms described above, HE can be difficult to diagnose. According to the majority of case reports of HE, one key finding leads to a corresponding diagnosis: abnormally elevated thyroid antibodies, namely thyroid peroxidase or thyroglobulin antibody, anti-TPO being the most common detected (62). The majority of cases occur in euthyroid or hypothyroid patients, even though HE can also occur in hyperthyroid patients (50, 63).

Abnormalities found in laboratory and imaging investigations are not pathognomonic but they may be useful in excluding other diagnoses (64). Even though cerebrospinal fluid analysis, electroencephalogram (EEG) and neuroimaging studies are not diagnostic, they may reveal some uncharacteristic changes (65). The most common abnormality identified in CSF analysis is elevated protein levels. In some cases, a mild lymphocytic pleocytosis can also be found (15, 66). Another helpful marker for the diagnosis of HE is the detection of anti-thyroid antibodies in CSF, which are present in the majority of cases (67). A literature review performed by Chong et al. proved that EEG is an useful tool in the diagnosis of HE, with abnormal EEG results being recorded in 98% of patients with HE (68). The main EEG finding consists in slow wave abnormalities, but epileptiform abnormalities, focal slowing, triphasic waves and photic stimulation induced discharges can also be found (69, 70). Brain MRI is usually normal, although in some cases, non-specific findings are observed, such as white matter changes, edema, atrophy, and ischemic lesions (71). Existing literature demonstrates that some of these abnormalities can be reversible after treatment (72). A recently published article reported the first description of conus medullaris involvement in HE, suggesting that the extension of MRI study to spinal cord may allow finding new pathological lesions useful in HE’s diagnosis (73).

Taking into consideration the wide spectrum of symptoms met in HE and the non-specific laboratory and imaging investigations, a proper differential diagnosis should be made. Infectious, metabolic, vascular, neoplastic, paraneoplastic, neurodegenerative, psychiatric and other autoimmune etiologies should be ruled out (74–76).

### 4.4. Treatment

Despite the severe clinical manifestations which can occur in HE, once the diagnosis is made and the right treatment is initiated, HE becomes a treatable and easily reversible cause of acute encephalopathy, with a good prognosis.

Given the rarity of the disease, treatment guidelines are not clearly established. Corticosteroid therapy is the treatment of choice, HE being also called “steroid responsive encephalopathy associated with autoimmune thyroiditis” (SREAT) (77). Treatment is generally initiated with methylprednisolone 500–1,000 mg intravenous for 3–7 days followed by oral prednisone 1–2 mg/kg/day, with a gradual tapering of steroid dose after the desired result is achieved (78). Depending on the case, corticosteroid therapy duration can vary from months to years (79). There is typically an improvement or complete resolution of the symptoms within a few months (5). In the event of disease recurrence or occurrence of side effects associated with steroids, other immunotherapies can be added, such as mycophenolate, azathioprine, cyclophosphamide, methotrexate, rituximab (80, 81). It is important to note that long-term immunomodulatory therapy does not come without risks – serious side effects are possible and clinical and laboratory parameters must be closely monitored on a regular basis (82). Other effective immunotherapies associated with a shorter duration of therapy and less side effects are represented by IVIG and plasma exchange.

#### 4.4.1. Intravenous immunoglobulin

**Figure 3:**
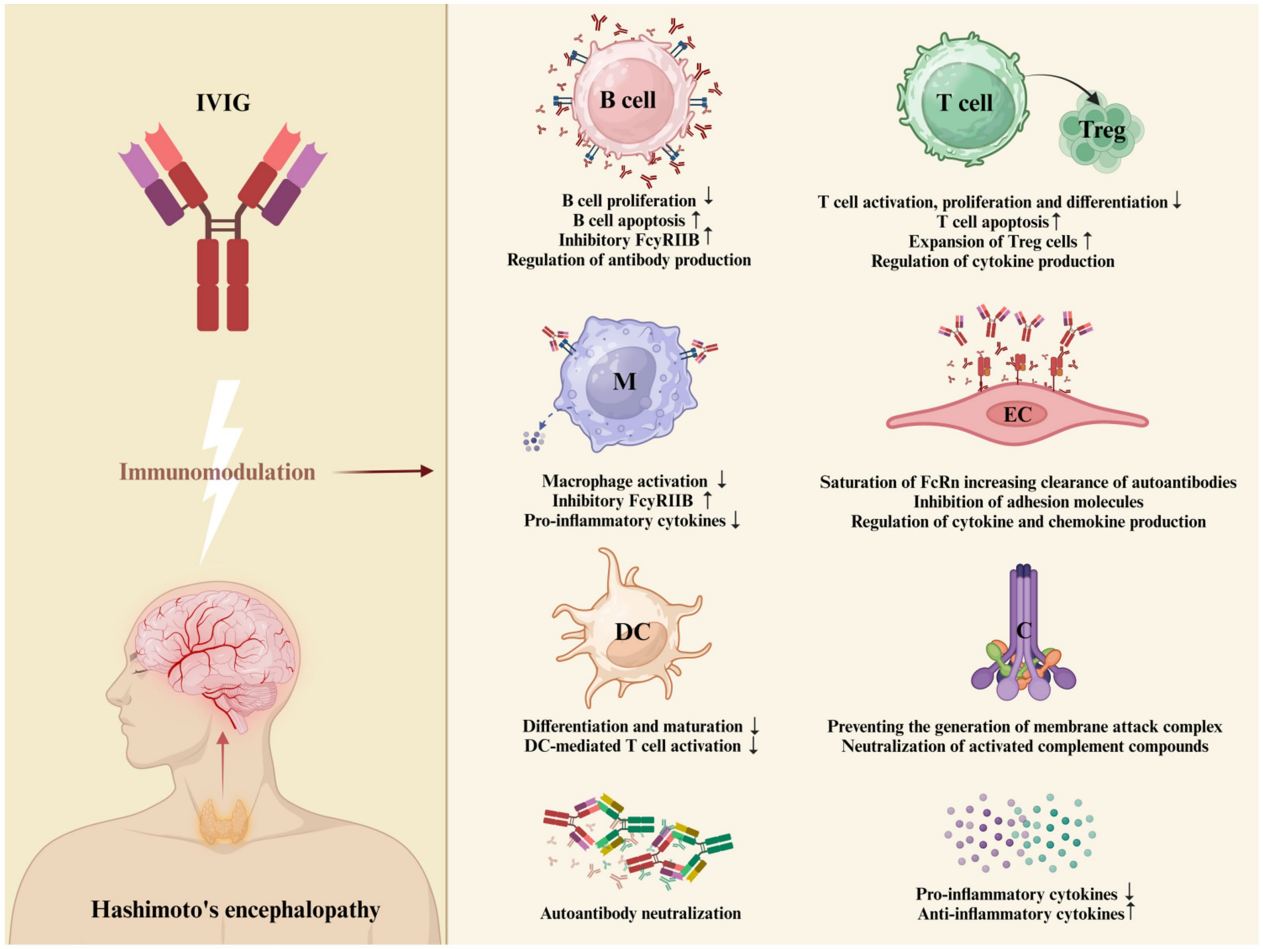
Proposed mechanism of action of IVIG in HE. IVIG employs multiple distinct mechanisms to exert its regulatory effects on various cellular and soluble constituents of the immune system. These mechanisms are not mutually exclusive and have the potential to collaboratively modulate the therapeutic outcomes. The proposed mechanism of action of IVIG in HE include: FcγR blockade leading to the potential inhibition of opsonized antigen binding, reduction in macrophage-secreted pro-inflammatory cytokines, and attenuation of granulocyte degranulation; stimulation of FcγRIIB to inhibit antibody production (84); intensified removal of pathogenic autoantibodies by saturating the FcRn or by IVIG binding to and neutralizing autoantibodies in serum (85); inhibition of T-cell proliferation and enhancing of the supressive properties of regulatory T (Treg) cells (86); prevention of membranolytic attack complex (MAC) generation and neutralization of C3a and C5a components; supressed production of pro-inflammatory cytokines, chemokines and adhesion molecules; stimulation of the production of anti-inflammatory cytokines (87); inhibition of differentiation and maturation of dendritic cells (84) (Created with BioRender.com). *B cell = B lymphocyte; M = macrophage; DC = dendritic cell; T cell = T lymphocyte; Treg = regulatory T cell; EC = endothelial cell; C = complement system.

Intravenous immunoglobulin is a concentrate of the pooled immunoglobulins obtained from at least 1,000 of healthy donors, prepared by using Cohn-Oncley procedure. Immunoglobulin is primarily composed of IgG, but it also contains various amounts of other proteins and auxiliary materials (83). The mechanisms by which IVIG has anti-inflammatory or immunomodulatory properties (Figure 3) have been difficult to define, but they were mainly attributed to blockade of the Fcγ receptor (FcγR) on immune cells, autoantibody neutralization by saturation of the neonatal Fc receptor (FcRn), inhibition of autoantibody production by stimulation of the Fc gamma receptor IIB (FcγRIIB), modulation of cytokine production and complement inhibition (84–89).

The use of intravenous immunoglobulin in the last 3 decades has revolutionized the treatment of previously untreatable conditions. The first time when IVIG were used for an autoimmune disease was in 1980 when Imbach et al. successfully used large intravenous doses of polyvalent Ig concentrate in children with acute, intermittent or chronic idiopathic thrombocytopenic purpura. A significant increase in platelet count was observed within 5 days in all patients, with variations in the subsequent course depending on the form of disease (90). This was just the first step in the success story of using IVIG preparations as an effective immunomodulatory therapy for a wide variety of conditions, including autoimmune or inflammatory diseases (91). The use of intravenous immunoglobulins in clinical neurology has been shown to be valuable in the treatment of new-onset or recurrent immune disorders as well as in chronic maintenance therapy (92). There is a strong evidence base for the use of IVIG therapy in Guillain-Barre syndrome, Chronic Inflammatory Demyelinating Polyneuropathy, multifocal motor neuropathy (93–95). In July 2021, based on the ProDERM study, U.S Food & Drug Administration (FDA) also approved a solution of IVIG for the treatment of adults with dermatomyositis (96). Additionally, based on controlled clinical trials, IVIG has shown to be effective in neurological conditions such as stiff-person syndrome, myasthenia gravis, inflammatory myopathies, multiple sclerosis, optic neuritis or autoimmune encephalitis (97, 98).

Intravenous immunoglobulin is generally considered a safe therapy, the majority of adverse effects associated to its administration being mild and transient. The occurrence of adverse effects depends on multiple factors, such as components of immunoglobulin products, rate of infusion and pacient-related risk factors (99). Depending on the time of occurrence, adverse effects can be immediate or delayed. The most frequent ones, representing more than 80% of intravenous immunoglobulin-induced adverse effects, are represented by flu-like symptoms, manifesting with fever, headache, chills, nausea, myalgia (100, 101). These symptoms are most often associated with rapid infusions and typically occur during the initial period of infusion. Other immediate adverse effects include dermatological reactions, chest tightness, dyspnea, vomiting, diarrhea, hypotension, tachycardia and anaphylactic reactions (102). Severe anaphylactic reactions are rare and they usually occur in IgA-deficient patients (103). The immediate adverse effects usually improve with the reduction of infusion rate or the temporary discontinuation of the infusion. Symptomatic therapy with analgesics, nonsteroidal anti-inflammatory drugs, antihistamines or glucocorticoids can also be used (102). Late adverse affects are rare but they can be severe or lethal. Thrombotic events, such as stroke, myocardial infarction and pulmonary embolism, can occur in patients being at high risk, due to the increased plasma viscosity induced by IVIG administration (104, 105). Renal impairment, another delayed side effect associated with intravenous immunoglobulin therapy, usually affects patients with preexisting renal dysfunction, diabetes, advanced age and dehydration. It can be avoided with a correct assessment of risk factors, proper pre-treatment hydration, urine output and kidney function monitoring and avoidance of sucro-stabilized immunoglobulin products which can induce renal failure by osmotic injury (106). Other delayed adverse affects are represented by pseuhohyponatremia, neutropenia, autoimmune hemolytic anemia, seizures, aseptic meningitis (107–111). Despite of all the possible adverse effects which can be associated with IVIG, it is still a well tolerated therapy with transient side effects, which changed the therapeutic approach in HE over the last decades.

The first case report of using IVIG for treating HE was published in 1998 by Wirkowski et al. They presented the case of a 82-year-old woman who developed neurological symptoms such as changes in mental status and multifocal neurological deficits in the presence of elevated titers of anti-thyroid antibodies. After an initial ineffective treatment with prednisone and methotrexate, an important clinical improvement was obtained with monthly courses of IVIG (17).

One interesting case reported by Jacob et al. in 2005 describes a 29-year-old woman who had multiple episodes of confusion and agitation over a period of 14 years, without a clear diagnosis. The initial hospital admission was in 1987 when cerebrospinal fluid examination identified pleocytosis and a presumed diagnosis of meningo-encephalitis was made. The patient had a good recovery with the antibiotic treatment but during the next years, the symptoms still persisted and she was also considered to have a psychiatric disorder. In 2003, another episode of confusion and drowsiness led to further investigations which identified an elevated thyroid peroxidase antibody titer. A remarkable response was obtained after a 5-day course of intravenous dexamethasone. In May 2004 the patient was re-admitted with the same symptoms but this time the confusion and agitation worsened even if another course of intravenous dexamethasone was administered. This was the moment when 400 mg/kg intravenous immunoglobulin was given, with a significant improvement within 12 h. The treatment had to be stopped because of a septicaemia resulted from an infected cannula site, but after a course of antibiotics the IVIG therapy was reinstated for 5 days with a complete recovery of the patient (16).

In the majority of cases (7, 8, 11–17), the initial treatment consisted in oral or intravenous corticotherapy, among with other immunosuppressive medication (e.g., methotrexate) or symptomatic medication (e.g., antiepileptic drugs). No improvement, partial improvement or full improvement but with the relapse of HE were identified. After intravenous immunoglobulin administration, a remarkable clinical response was obtained, either consisting in a full recovery in a short period of time without the need of using more medication, or a partial recovery with the need of administration of another rounds of IVIG among tapering of steroid therapy.

In some cases, IVIG was used as the first-line therapy, either alone or in combination with steroid therapy (5, 6, 9, 10). These cases were published in the last 7 years, so the initial use of IVIG may be the result of the data found in literature which sustains the beneficial effects of IVIG in the treatment of HE.

Based on our literature research, the first case-report about the use of IVIG as a first-line treatment was published in 2017 by Zhu et al. They presented the case of a 54-year-old woman who suffered from progressive cognitive decline and was initially treated with acyclovir for a suspicion of viral encephalitis. Four days later the patient went into a coma and 3 days of IVIG therapy was initiated, followed by dexamethasone. The outcome was remarkable, with a fully recovery from coma. However, at 3 months follow-up, the patient’s memory deficits did not completely recover. What was also interesting about this case, beside the IVIG ability to wake up a patient out of a coma, was the identification of antibodies against α-amino-3-hydroxy-5-methyl-4-isoxazole-propionic acid receptor (AMPAR) both in serum and cerebrospinal fluid. This was also the first case report on the detection of anti-AMPAR antibodies in HE (10). AMPAR is a subtype of glutamate receptor which mediates fast excitatory synaptic transmission in the central nervous system, being involved in synaptic plasticity, learning and memory (112). They were initially described by Lai et al. in 10 patients with limbic encephalitis (113) and multiple studies concluded that anti-AMPAR can be associated with a coexisting neoplasia (114), which was ruled out in Zhu et al.’s case. Zhu et al. speculated that in HE, the production of anti-AMPAR can be the result of immune dysfunction in the brain induced by the elevated levels of anti-thyroid antibodies (10).

Laycock et al. described the case of a 28-year-old woman who received a diagnosis of autoimmune thyroiditis at 20 years old. In the last years she was suffering of chronic fatigue, poor concentration, cognitive decline, symptoms which made her unable to sustain employment. Blood tests showed an adequate thyroxin replacement. After the diagnosis of HE was made, based on elevated levels of anti-TPO both in blood and cerebrospinal fluid, they had to choose the adequate treatment. Her BMI was 35.7 kg/m^2^ so there were some concerns raised about the metabolic adverse effects of steroid therapy. As a result, IVIG was used as the first-line treatment, with significant improvement in general intellectual functioning (9). This case shows the importance of tailoring the treatment to the patient.

A case reported by Alladin et al. in 2022 focused on a rare presentation of HE, characterized by rapidly progressive dementia with irreversible cerebral damage which rendered steroid therapy and intravenous immunoglobulin ineffective. It was the case of a 47-year-old woman who had a 2-year history of progressive decline in memory, emotional lability, insomnia, agitation, generalized tonic–clonic seizures, global rigidity with declined mobility which made her bedbound. Brain imaging investigations identified vascular abnormalities consistent with vasculitis of the large and medium arteries, among severe atrophy of the caudate and temporal lobes which were probably the result of a chronic cerebral inflammation caused by a long-lasting occult form of HE. The patient received two courses of steroid therapy (1 g methylprednisolone) with limited response, followed by a course of intravenous immunoglobulin, which also did not lead to a sensible change in the clinical condition (4). The current case shows the importance of HE early diagnosis, despite the versatile clinical presentations, and of timely management with immunosuppressive therapy in order to prevent permanent damage of the central nervous system.

In the majority of the selected case-reports, IVIG was associated with a good outcome, sometimes even with dramatic improvements in patient’s status. It was generally used as a second-line therapy, but the 3 cases when IVIG was used as a first-line therapy may offer new perspectives about the initial treatment approach in HE.

## 5. Conclusion

Hashimoto’s encephalopathy still remains a challenging disease, due to the variety of clinical manifestations, poorly understood pathogenesis, non-specific laboratory and imaging abnormalities and lack of treatment guidelines. Early diagnosis and treatment are essential for avoiding irreversible damage.

Steroids represent the current standard treatment of HE but steroid-resistant HE cases have also been reported. The non-responsive cases to the corticosteroid treatment, or those suffering from severe adverse effects, can receive other immunomodulatory therapies, such as mycophenolate, azathioprine, cyclophosphamide, methotrexate or rituximab. These therapies can also have serious side effects, so there is a need for a safer alternative treatment option.

In last years, IVIG therapy proved its utility in HE’s treatment, being a well tolerated treatment associated with remarkable improvement in patient’s status. Further research is still needed in order to define the optimal treatment protocol for HE and to establish if IVIG can also be used as a first-line therapy, alone or in combination with steroids.

## Data availability statement

The original contributions presented in the study are included in the article/supplementary material, further inquiries can be directed to the corresponding authors.

## Author contributions

VŞ: data curation, methodology, supervision, and conceptualization. MC: methodology, writing – original draft, supervision, and conceptualization. AA: data curation, investigation, and writing – original draft. CL: writing – review and editing, methodology, and supervision. AC: investigation and writing – review and editing. OS: data curation and writing – original draft. RH: data curation, writing-review and editing. LŞ: investigation, methodology, supervision, and conceptualization. All authors contributed to the article and approved the submitted version.

## Conflict of interest

The authors declare that the research was conducted in the absence of any commercial or financial relationships that could be construed as a potential conflict of interest.

## Publisher’s note

All claims expressed in this article are solely those of the authors and do not necessarily represent those of their affiliated organizations, or those of the publisher, the editors and the reviewers. Any product that may be evaluated in this article, or claim that may be made by its manufacturer, is not guaranteed or endorsed by the publisher.

## Figures and Tables

**Table:** 

HE	Hashimoto’s Encephalopathy
SREAT	Steroid responsive encephalopathy associated with autoimmune thyroiditis
CSF	Cerebrospinal fluid
IVIG	Intravenous immunoglobulin
TSH	Thyroid stimulating hormone
anti-TPO	Anti-thyroid peroxidase
anti-Tg	Anti-thyroglobulin
CT	Computer tomography
MRI	Magnetic resonance imaging
HT	Hashimoto’s thyroiditis
APS	Autoimmune polyendocrine syndromes
i.v	Intravenous
TRH	Thyrotropin-releasing hormone
NH2-α-enolase Ab	Antibodies against amino terminal domain of α-enolase
AKRIAI	Aldehyde reductase-I;
DDAHI	Dimethylargininase-I
anti-NMDArE	Anti-N methyl D-aspartate receptor encephalitis
IgG4-RD	IgG4-related disease
EEG	Electroencephalogram
Fc	Fragment crystallizable region
Ig	Immunoglobulin
FcγR	Fc gamma receptor
FcRn	Neonatal Fc receptor
FcγRIIB	Fc gamma receptor IIB
AMPAR	α-Amino-3-hydroxy-5-methyl-4-isoxazole-propionic acid receptor
